# Supplementary material for: Gene co-expression network analysis in human spinal cord highlights mechanisms underlying amyotrophic lateral sclerosis susceptibility
Source: Sci Rep. 2021 Mar 11;11:5748. doi: 10.1038/s41598-021-85061-4 (PMC7970949; doi:10.1038/s41598-021-85061-4)
Supplement: Supplementary file 1 — Supplementary Information 1. [file 41598_2021_85061_MOESM1_ESM.pdf]

# Gene co-expression network analysis in human spinal cord highlights mechanisms underlying amyotrophic lateral sclerosis susceptibility

Jerry C. Wang, Gokul Ramaswami, Daniel H. Geschwind

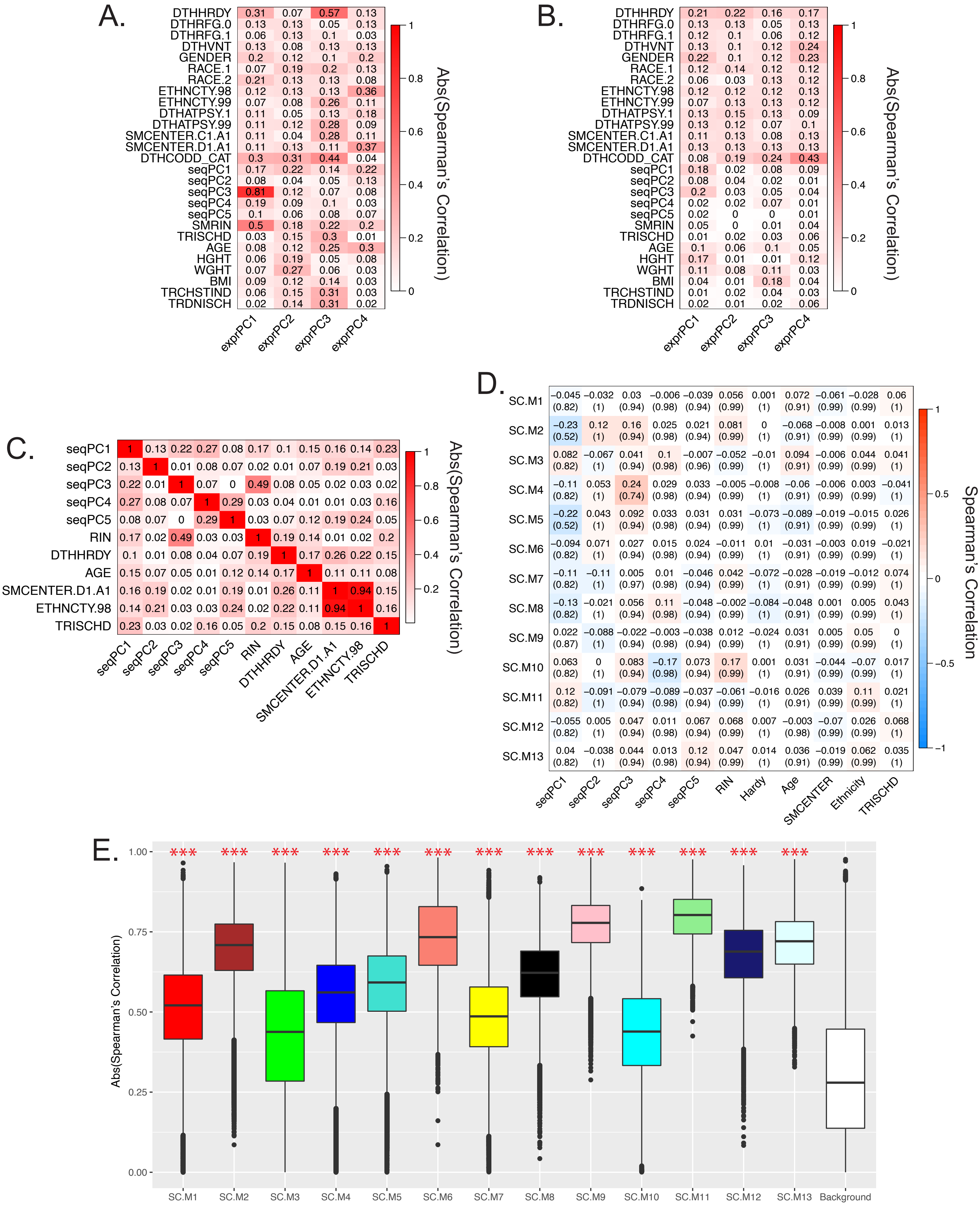
A.
B.
DTHHRDY
DTHRFG.0
DTHRFG.1
DTHVNT
GENDER
RACE.1
RACE.2
ETHNCTY.98
ETHNCTY.99
DTHATPSY.1
DTHATPSY.99
SMCENTER.C1.A1
SMCENTER.D1.A1
DTHCODD_CAT
seqPC1
seqPC2
seqPC3
seqPC4
seqPC5
SMRIN
TRISCHD
AGE
HGHT
WGHT
BMI
TRCHSTIND
TRDNISCH
exprPC1
exprPC2
exprPC3
exprPC4
Abs(Spearman's Correlation)
C.
seqPC1
seqPC2
seqPC3
seqPC4
seqPC5
RIN
DTHHRDY
AGE
SMCENTER.D1.A1
ETHNCTY.98
TRISCHD
Abs(Spearman's Correlation)
D.
SC.M1
SC.M2
SC.M3
SC.M4
SC.M5
SC.M6
SC.M7
SC.M8
SC.M9
SC.M10
SC.M11
SC.M12
SC.M13
seqPC1
seqPC2
seqPC3
seqPC4
seqPC5
RIN
Hardy
Age
SMCENTER
Ethnicity
TRISCHD
Spearman's Correlation
E.
Abs(Spearman's Correlation)
SC.M1
SC.M2
SC.M3
SC.M4
SC.M5
SC.M6
SC.M7
SC.M8
SC.M9
SC.M10
SC.M11
SC.M12
SC.M13
Background

**Supplementary Figure S1.** Correction for technical and biological drivers of expression variance in the GTEX human spinal cord dataset.

(A) Spearman's correlations between the top four expression principal components (exprPCs1-4) and technical and biological covariates. RIN is RNA Integrity Number. DTHHRDY indicates Hardy's Death Classification which measures time from first onset of symptoms to death. TRISCHD indicates ischemic time as defined by the time of death to when tissue was fixed or frozen. Complete code annotations can be found in the GTEx Portal v7.

(B) Same as (A), except using the covariate-corrected human spinal cord expression matrix.

(C) Pairwise Spearman's correlations between biological and technical covariates in the human spinal cord dataset to check for covariate collinearity.

(D) Spearman's correlations of module eigengenes to traits in the covariate-corrected human spinal cord dataset. Significance was assessed using Student's t-test.

(E) Pairwise Spearman's correlations of 1,000 randomly sampled genes from each module and a background set. Significance of difference between each module with the background set was assessed using Student's t-test. *** (FDR corrected p-value < 0.001).

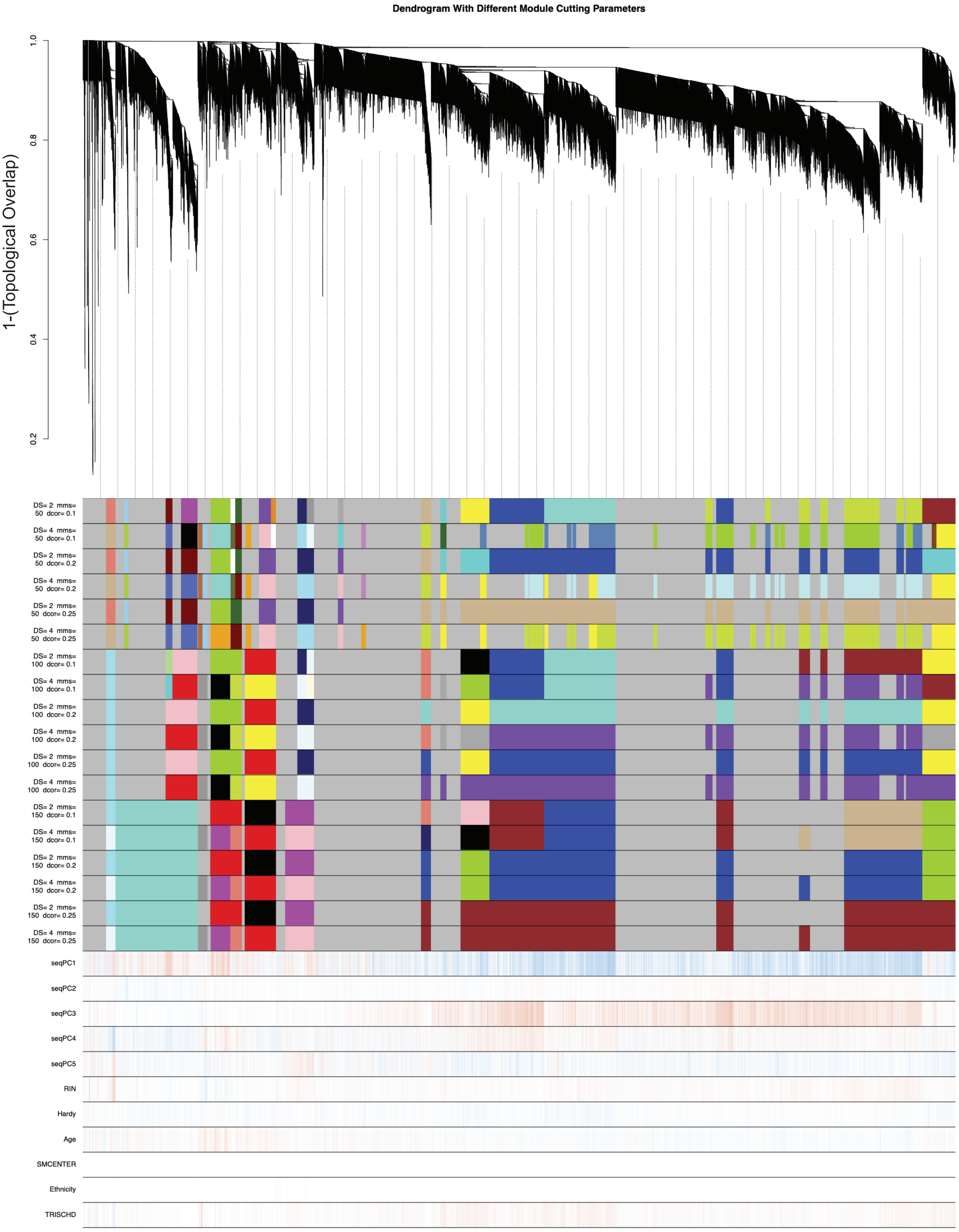

Dendrogram With Different Module Cutting Parameters
1-(Topological Overlap)
0.2
0.4
0.6
0.8
1.0
DS= 2 mms= 50 dcor= 0.1
DS= 4 mms= 50 dcor= 0.1
DS= 2 mms= 50 dcor= 0.2
DS= 4 mms= 50 dcor= 0.2
DS= 2 mms= 50 dcor= 0.25
DS= 4 mms= 50 dcor= 0.25
DS= 2 mms= 100 dcor= 0.1
DS= 4 mms= 100 dcor= 0.1
DS= 2 mms= 100 dcor= 0.2
DS= 4 mms= 100 dcor= 0.2
DS= 2 mms= 100 dcor= 0.25
DS= 4 mms= 100 dcor= 0.25
DS= 2 mms= 150 dcor= 0.1
DS= 4 mms= 150 dcor= 0.1
DS= 2 mms= 150 dcor= 0.2
DS= 4 mms= 150 dcor= 0.2
DS= 2 mms= 150 dcor= 0.25
DS= 4 mms= 150 dcor= 0.25
seqPC1
seqPC2
seqPC3
seqPC4
seqPC5
RIN
Hardy
Age
SMCENTER
Ethnicity
TRISCHD

**Supplementary Figure S2.** Optimization of tree cutting parameters to generate co-expression modules.

Dendrogram of the topological overlap matrix for gene co-expression dissimilarity. Co-expression module assignments are shown in each color track. Various permutations of minimum module size (mms), module merging (dcor), and deep split parameters (DS) were tested. Spearman correlations for each gene to biological and technical covariates are shown below the color tracks (Blue: Negative; Red: Positive).

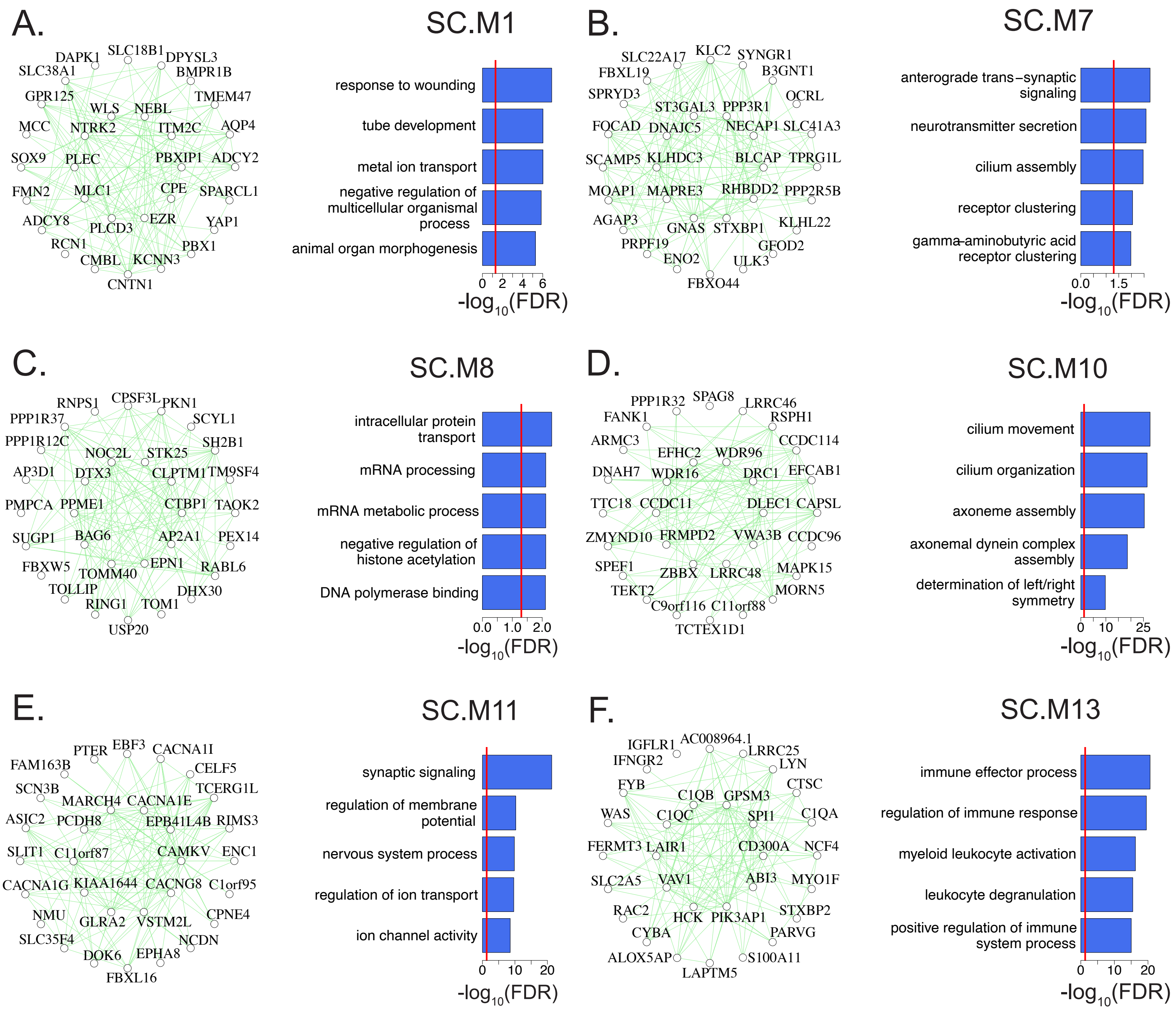
A.
SC.M1
DAPK1 SLC18B1 DPYSL3
SLC38A1 BMPR1B
GPR125 WLS NEBL TMEM47
MCC NTRK2 ITM2C AQP4
SOX9 PLEC PBXIP1 ADCY2
FMN2 MLC1 CPE SPARCL1
ADCY8 PLCD3 EZR YAP1
RCN1 PBX1
CMBL KCNN3
CNTN1
response to wounding
tube development
metal ion transport
negative regulation of multicellular organismal process
animal organ morphogenesis
0 2 4 6
-log10(FDR)
B.
SC.M7
SLC22A17 KLC2 SYNGR1
FBXL19 B3GNT1
SPRYD3 OCRL
ST3GAL3 PPP3R1
FOCAD DNAJC5 NECAP1 SLC41A3
SCAMP5 KLHDC3 BLCAP TPRG1L
MOAP1 MAPRE3 RHBDD2 PPP2R5B
AGAP3 GNAS STXBP1 KLHL22
PRPF19 GFOD2
ENO2 ULK3
FBXO44
anterograde trans−synaptic signaling
neurotransmitter secretion
cilium assembly
receptor clustering
gamma-aminobutyric acid receptor clustering
0.0 1.5
-log10(FDR)
C.
SC.M8
RNPS1 CPSF3L PKN1
PPP1R37 SCYL1
PPP1R12C NOC2L STK25 SH2B1
AP3D1 DTX3 CLPTM1 TM9SF4
PMPCA PPME1 CTBP1 TAOK2
SUGP1 BAG6 AP2A1 PEX14
FBXW5 TOMM40 EPN1 RABL6
TOLLIP DHX30
RING1 TOM1
USP20
intracellular protein transport
mRNA processing
mRNA metabolic process
negative regulation of histone acetylation
DNA polymerase binding
0.0 1.0 2.0
-log10(FDR)
D.
SC.M10
PPP1R32 SPAG8 LRRC46
FANK1 RSPH1
ARMC3 EFHC2 WDR96 CCDC114
DNAH7 WDR16 DRC1 EFCAB1
TTC18 CCDC11 DLEC1 CAPSL
ZMYND10 FRMPD2 VWA3B CCDC96
SPEF1 ZBBX LRRC48 MAPK15
TEKT2 MORN5
C9orf116 C11orf88
TCTEX1D1
cilium movement
cilium organization
axoneme assembly
axonemal dynein complex assembly
determination of left/right symmetry
0 10 25
-log10(FDR)
E.
SC.M11
PTER EBF3 CACNA1I
FAM163B CELF5
SCN3B TCERG1L
MARCH4 CACNA1E
ASIC2 PCDH8 EPB41L4B RIMS3
SLIT1 C11orf87 CAMKV ENC1
CACNA1G KIAA1644 CACNG8 C1orf95
NMU GLRA2 VSTM2L CPNE4
SLC35F4 NCDN
DOK6 EPHA8
FBXL16
synaptic signaling
regulation of membrane potential
nervous system process
regulation of ion transport
ion channel activity
0 10 20
-log10(FDR)
F.
SC.M13
IGFLR1 AC008964.1 LRRC25
IFNGR2 LYN
FYB CTSC
C1QB GPSM3
WAS C1QC SPI1 C1QA
FERMT3 LAIR1 CD300A NCF4
SLC2A5 VAV1 ABI3 MYO1F
RAC2 STXBP2
HCK PIK3AP1
CYBA PARVG
ALOX5AP S100A11
LAPTM5
immune effector process
regulation of immune response
myeloid leukocyte activation
leukocyte degranulation
positive regulation of immune system process
0 10 20
-log10(FDR)

**Supplementary Figure S3.** GO term annotations and top hubs of co-expression modules not displayed in the main figures.

(A, B, C, D, E, F) Top 30 hub genes and 300 connections for SC.M1, SC.M7, SC.M8, SC.M10, SC.M11, and SC.M13 respectively (left panels). Top 5 enriched GO terms for each of the respective modules (right panels). Red lines mark an FDR corrected p-value threshold of 0.05.

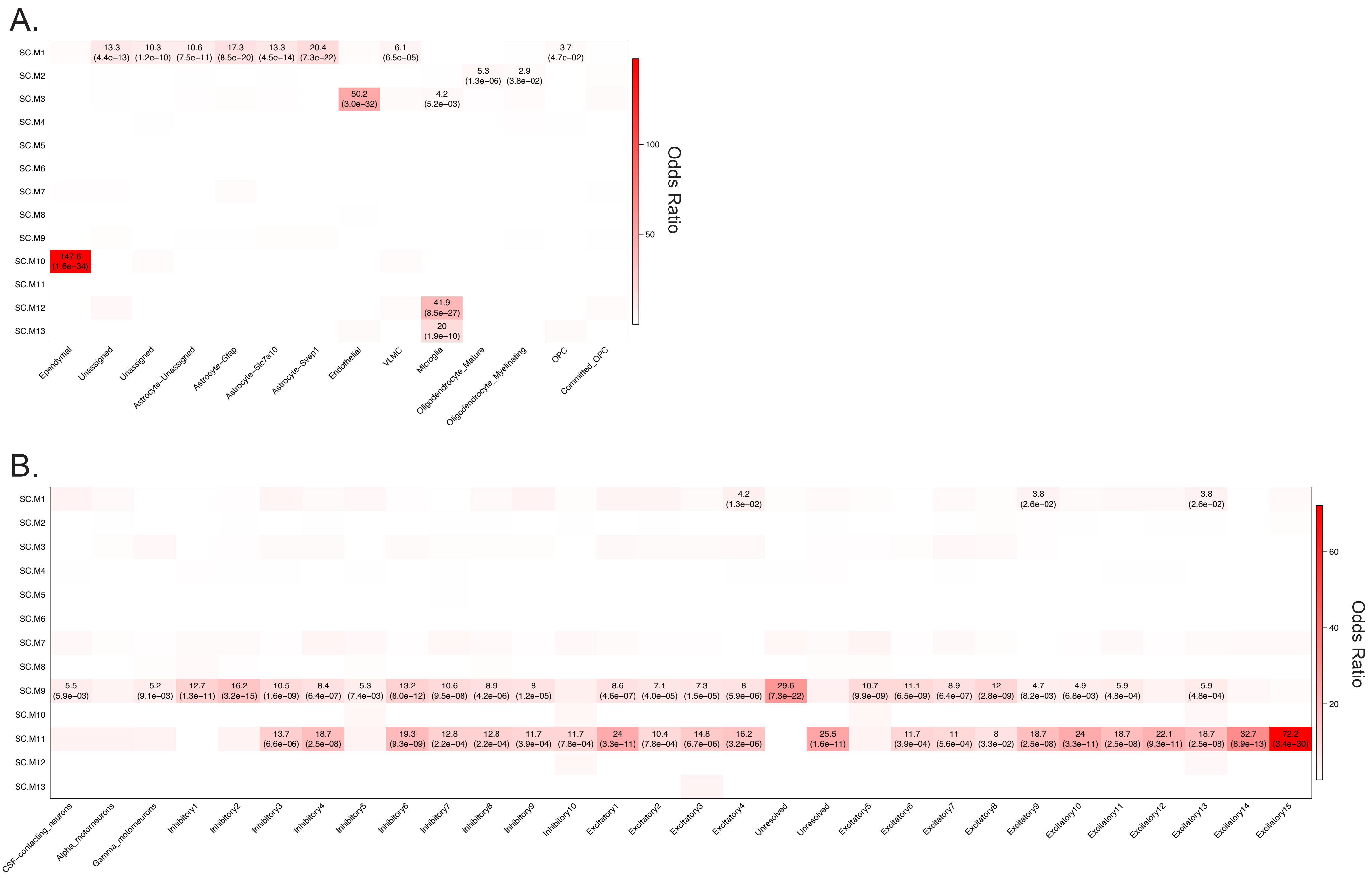
A.
SC.M1
SC.M2
SC.M3
SC.M4
SC.M5
SC.M6
SC.M7
SC.M8
SC.M9
SC.M10
SC.M11
SC.M12
SC.M13
13.3 (4.4e−13)
10.3 (1.2e−10)
10.6 (7.5e−11)
17.3 (8.5e−20)
13.3 (4.5e−14)
20.4 (7.3e−22)
6.1 (6.5e−05)
3.7 (4.7e−02)
5.3 (1.3e−06)
2.9 (3.8e−02)
50.2 (3.0e−32)
4.2 (5.2e−03)
147.6 (1.6e−34)
41.9 (8.5e−27)
20 (1.9e−10)
Ependymal
Unassigned
Unassigned
Astrocyte−Unassigned
Astrocyte−Gfap
Astrocyte−Slc7a10
Astrocyte−Svep1
Endothelial
VLMC
Microglia
Oligodendrocyte_Mature
Oligodendrocyte_Myelinating
OPC
Committed_OPC
100
50
Odds Ratio
B.
SC.M1
SC.M2
SC.M3
SC.M4
SC.M5
SC.M6
SC.M7
SC.M8
SC.M9
SC.M10
SC.M11
SC.M12
SC.M13
4.2 (1.3e−02)
3.8 (2.6e−02)
3.8 (2.6e−02)
5.5 (5.9e−03)
5.2 (9.1e−03)
12.7 (1.3e−11)
16.2 (3.2e−15)
10.5 (1.6e−09)
8.4 (6.4e−07)
5.3 (7.4e−03)
13.2 (8.0e−12)
10.6 (9.5e−08)
8.9 (4.2e−06)
8 (1.2e−05)
8.6 (4.6e−07)
7.1 (4.0e−05)
7.3 (1.5e−05)
8 (5.9e−06)
29.6 (7.3e−22)
10.7 (9.9e−09)
11.1 (6.5e−09)
8.9 (6.4e−07)
12 (2.8e−09)
4.7 (8.2e−03)
4.9 (6.8e−03)
5.9 (4.8e−04)
5.9 (4.8e−04)
13.7 (6.6e−06)
18.7 (2.5e−08)
19.3 (9.3e−09)
12.8 (2.2e−04)
12.8 (2.2e−04)
11.7 (3.9e−04)
11.7 (7.8e−04)
24 (3.3e−11)
10.4 (7.8e−04)
14.8 (6.7e−06)
16.2 (3.2e−06)
25.5 (1.6e−11)
11.7 (3.9e−04)
11 (5.6e−04)
8 (3.3e−02)
18.7 (2.5e−08)
24 (3.3e−11)
18.7 (2.5e−08)
22.1 (9.3e−11)
18.7 (2.5e−08)
32.7 (8.9e−13)
72.2 (3.4e−30)
CSF−contacting_neurons
Alpha_motorneurons
Gamma_motorneurons
Inhibitory1
Inhibitory2
Inhibitory3
Inhibitory4
Inhibitory5
Inhibitory6
Inhibitory7
Inhibitory8
Inhibitory9
Inhibitory10
Excitatory1
Excitatory2
Excitatory3
Excitatory4
Unresolved
Unresolved
Excitatory5
Excitatory6
Excitatory7
Excitatory8
Excitatory9
Excitatory10
Excitatory11
Excitatory12
Excitatory13
Excitatory14
Excitatory15
60
40
20
Odds Ratio

**Supplementary Figure S4.** Enrichment of cell-type markers from mouse spinal cord within co-expression modules.

(A) Enrichments of glial cell-type markers from mouse spinal cord within co-expression modules. FDR corrected enrichment p-values < 0.05 are shown within the parentheses.

(B) Same as (A), except using neuronal cell-type markers.

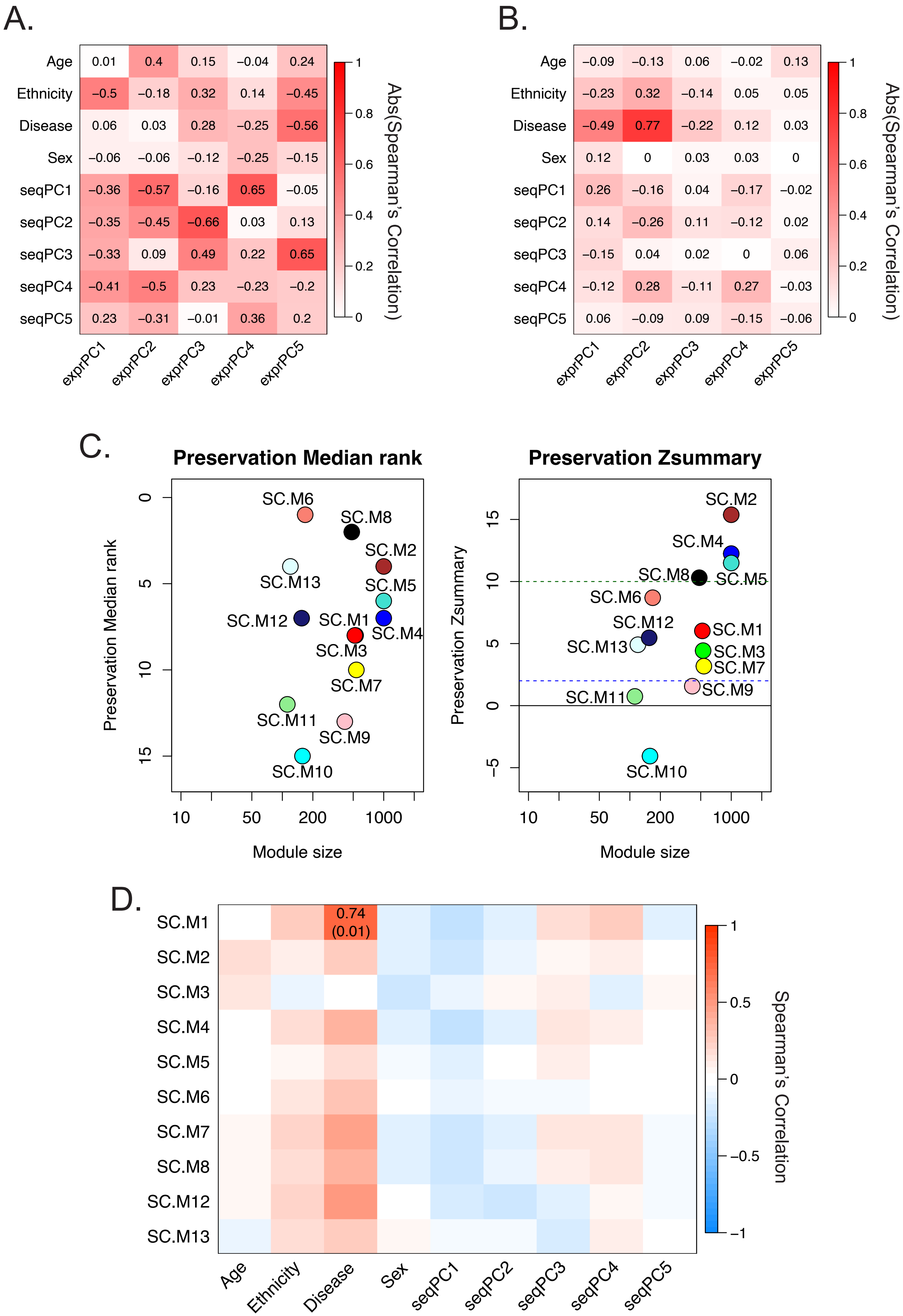
A.
Age 0.01 0.4 0.15 −0.04 0.24
Ethnicity −0.5 −0.18 0.32 0.14 −0.45
Disease 0.06 0.03 0.28 −0.25 −0.56
Sex −0.06 −0.06 −0.12 −0.25 −0.15
seqPC1 −0.36 −0.57 −0.16 0.65 −0.05
seqPC2 −0.35 −0.45 −0.66 0.03 0.13
seqPC3 −0.33 0.09 0.49 0.22 0.65
seqPC4 −0.41 −0.5 0.23 −0.23 −0.2
seqPC5 0.23 −0.31 −0.01 0.36 0.2
exprPC1 exprPC2 exprPC3 exprPC4 exprPC5
1 0.8 0.6 0.4 0.2 0
Abs(Spearman's Correlation)
B.
Age −0.09 −0.13 0.06 −0.02 0.13
Ethnicity −0.23 0.32 −0.14 0.05 0.05
Disease −0.49 0.77 −0.22 0.12 0.03
Sex 0.12 0 0.03 0.03 0
seqPC1 0.26 −0.16 0.04 −0.17 −0.02
seqPC2 0.14 −0.26 0.11 −0.12 0.02
seqPC3 −0.15 0.04 0.02 0 0.06
seqPC4 −0.12 0.28 −0.11 0.27 −0.03
seqPC5 0.06 −0.09 0.09 −0.15 −0.06
exprPC1 exprPC2 exprPC3 exprPC4 exprPC5
1 0.8 0.6 0.4 0.2 0
Abs(Spearman's Correlation)
C.
Preservation Median rank
Preservation Median rank
0 5 10 15
SC.M6
SC.M8
SC.M2
SC.M13
SC.M5
SC.M12
SC.M1
SC.M4
SC.M3
SC.M7
SC.M11
SC.M9
SC.M10
10 50 200 1000
Module size
Preservation Zsummary
Preservation Zsummary
15 10 5 0 −5
SC.M2
SC.M4
SC.M8
SC.M5
SC.M6
SC.M12
SC.M1
SC.M13
SC.M3
SC.M7
SC.M11
SC.M9
SC.M10
10 50 200 1000
Module size
D.
SC.M1
SC.M2
SC.M3
SC.M4
SC.M5
SC.M6
SC.M7
SC.M8
SC.M12
SC.M13
0.74
(0.01)
Age
Ethnicity
Disease
Sex
seqPC1
seqPC2
seqPC3
seqPC4
seqPC5
1 0.5 0 −0.5 −1
Spearman's Correlation

**Supplementary Figure S5.** Covariate correction, module preservation, and module-trait correlations in postmortem human ALS spinal cord.

(A) Spearman's correlations between the top five expression principal components (exprPCs1-5) and technical and biological covariates.

(B) Same as (A), except using the covariate-corrected expression matrix.

(C) Module preservation analysis was used to calculate the $Z_{summary}$ statistic for each module in postmortem human ALS spinal cord.

(D) Spearman's correlations of module eigengenes to traits correlated in postmortem human ALS spinal cord. Significance was assessed using Student's t-test. FDR corrected enrichment p-values < 0.05 are shown within the parentheses.

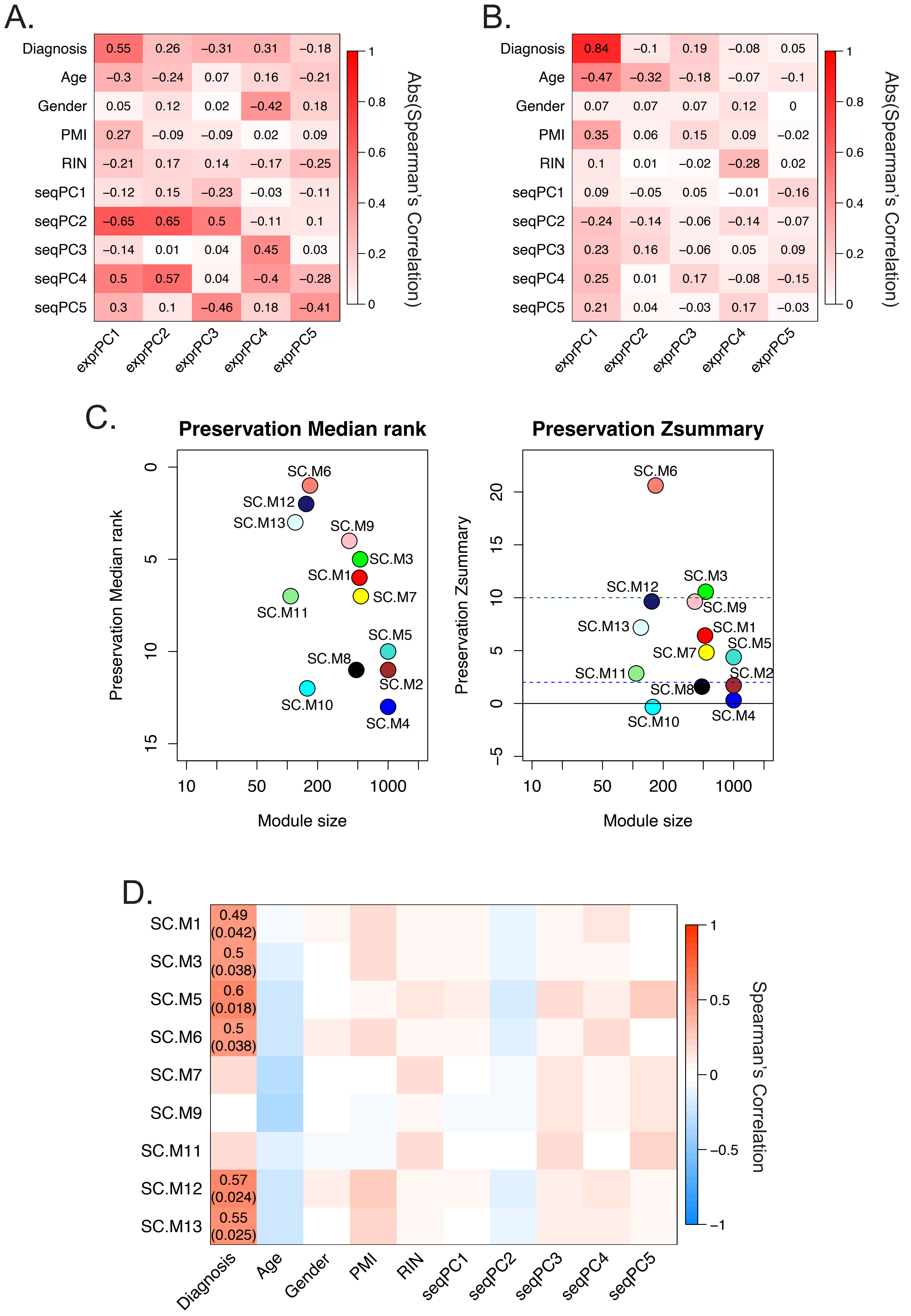

A.
Diagnosis 0.55 0.26 −0.31 0.31 −0.18
Age −0.3 −0.24 0.07 0.16 −0.21
Gender 0.05 0.12 0.02 −0.42 0.18
PMI 0.27 −0.09 −0.09 0.02 0.09
RIN −0.21 0.17 0.14 −0.17 −0.25
seqPC1 −0.12 0.15 −0.23 −0.03 −0.11
seqPC2 −0.65 0.65 0.5 −0.11 0.1
seqPC3 −0.14 0.01 0.04 0.45 0.03
seqPC4 0.5 0.57 0.04 −0.4 −0.28
seqPC5 0.3 0.1 −0.46 0.18 −0.41
exprPC1 exprPC2 exprPC3 exprPC4 exprPC5
Abs(Spearman's Correlation)
B.
Diagnosis 0.84 −0.1 0.19 −0.08 0.05
Age −0.47 −0.32 −0.18 −0.07 −0.1
Gender 0.07 0.07 0.07 0.12 0
PMI 0.35 0.06 0.15 0.09 −0.02
RIN 0.1 0.01 −0.02 −0.28 0.02
seqPC1 0.09 −0.05 0.05 −0.01 −0.16
seqPC2 −0.24 −0.14 −0.06 −0.14 −0.07
seqPC3 0.23 0.16 −0.06 0.05 0.09
seqPC4 0.25 0.01 0.17 −0.08 −0.15
seqPC5 0.21 0.04 −0.03 0.17 −0.03
exprPC1 exprPC2 exprPC3 exprPC4 exprPC5
Abs(Spearman's Correlation)
C.
Preservation Median rank
Preservation Zsummary
Module size
SC.M1 SC.M2 SC.M3 SC.M4 SC.M5 SC.M6 SC.M7 SC.M8 SC.M9 SC.M10 SC.M11 SC.M12 SC.M13
D.
SC.M1 0.49 (0.042)
SC.M3 0.5 (0.038)
SC.M5 0.6 (0.018)
SC.M6 0.5 (0.038)
SC.M7
SC.M9
SC.M11
SC.M12 0.57 (0.024)
SC.M13 0.55 (0.025)
Diagnosis Age Gender PMI RIN seqPC1 seqPC2 seqPC3 seqPC4 seqPC5
Spearman's Correlation

**Supplementary Figure S6**. Covariate correction, module preservation, and module trait correlations in presymptomatic motor neurons from human ALS spinal cord.

(A) Same as Supplementary Fig. S5a except using a dataset of presymptomatic motor neurons in the human ALS spinal cord. RIN is RNA Integrity Number. PMI is postmortem interval.

(B) Same as Supplementary Fig. S5b except using the covariate-corrected expression matrix generated from presymptomatic motor neurons in the human ALS spinal cord.

(C) Same as Supplementary Fig. S5c except module preservation was assessed in presymptomatic motor neurons from human ALS spinal cord dataset.

(D) Same as Supplementary Fig. S5d except using a dataset of presymptomatic motor neurons in the human ALS spinal cord. FDR corrected enrichment p-values < 0.05 are shown within the parentheses.

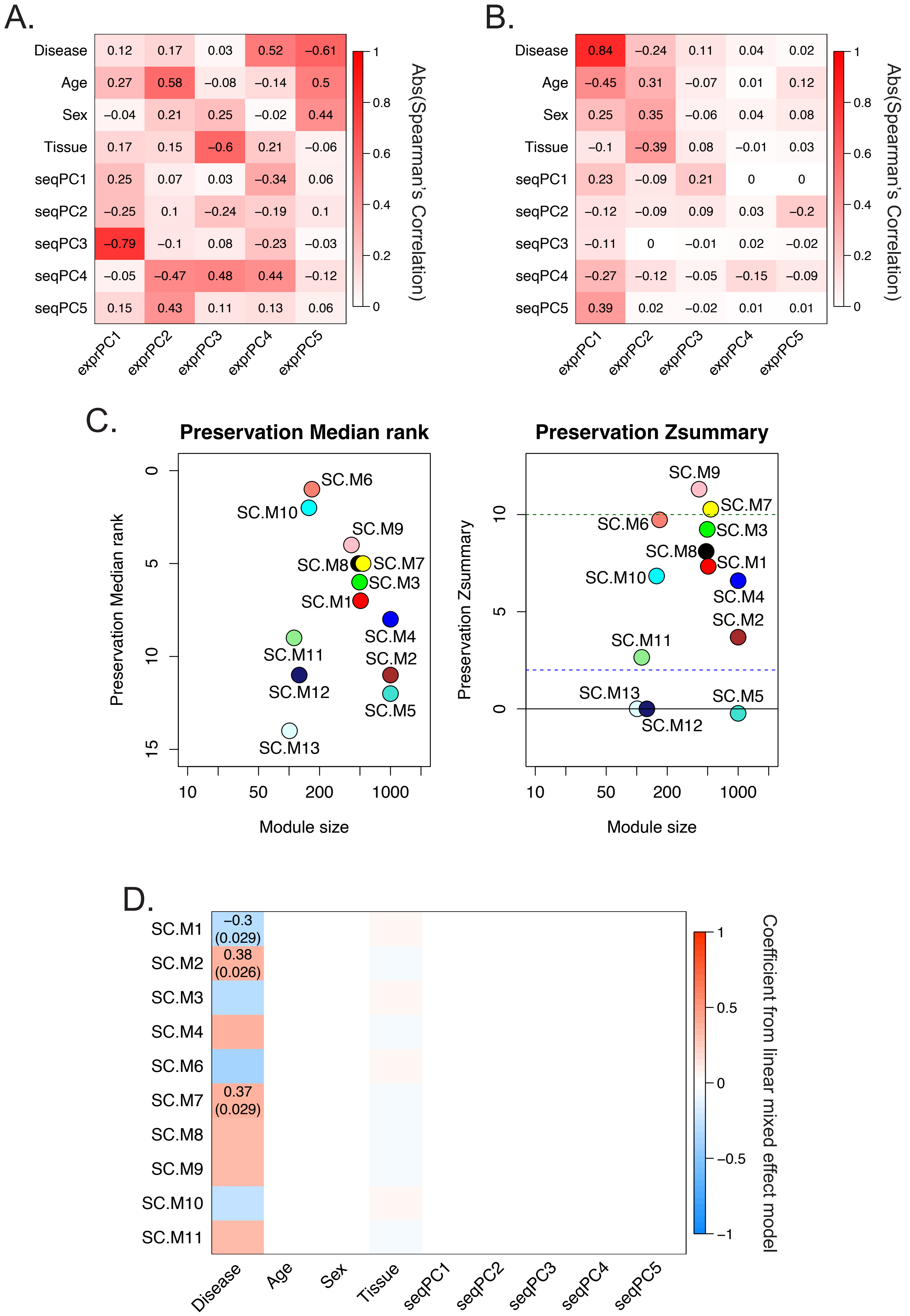
A.
Disease 0.12 0.17 0.03 0.52 −0.61
Age 0.27 0.58 −0.08 −0.14 0.5
Sex −0.04 0.21 0.25 −0.02 0.44
Tissue 0.17 0.15 −0.6 0.21 −0.06
seqPC1 0.25 0.07 0.03 −0.34 0.06
seqPC2 −0.25 0.1 −0.24 −0.19 0.1
seqPC3 −0.79 −0.1 0.08 −0.23 −0.03
seqPC4 −0.05 −0.47 0.48 0.44 −0.12
seqPC5 0.15 0.43 0.11 0.13 0.06
exprPC1 exprPC2 exprPC3 exprPC4 exprPC5
Abs(Spearman's Correlation)
B.
Disease 0.84 −0.24 0.11 0.04 0.02
Age −0.45 0.31 −0.07 0.01 0.12
Sex 0.25 0.35 −0.06 0.04 0.08
Tissue −0.1 −0.39 0.08 −0.01 0.03
seqPC1 0.23 −0.09 0.21 0 0
seqPC2 −0.12 −0.09 0.09 0.03 −0.2
seqPC3 −0.11 0 −0.01 0.02 −0.02
seqPC4 −0.27 −0.12 −0.05 −0.15 −0.09
seqPC5 0.39 0.02 −0.02 0.01 0.01
exprPC1 exprPC2 exprPC3 exprPC4 exprPC5
Abs(Spearman's Correlation)
C.
Preservation Median rank
Preservation Zsummary
Module size
SC.M1 SC.M2 SC.M3 SC.M4 SC.M5 SC.M6 SC.M7 SC.M8 SC.M9 SC.M10 SC.M11 SC.M12 SC.M13
D.
−0.3 (0.029)
0.38 (0.026)
0.37 (0.029)
Disease Age Sex Tissue seqPC1 seqPC2 seqPC3 seqPC4 seqPC5
Coefficient from linear mixed effect model

**Supplementary Figure S7.** Covariate correction, module preservation, and module-trait correlations in iPSC-derived motor neurons from ALS8 patients.

(A) Same as Supplementary Fig. S5a except using a dataset of iPSC-derived motor neurons from ALS8 patients.

(B) Same as Supplementary Fig. S5b except using the covariate-corrected expression matrix generated from iPSC-derived motor neurons from ALS8 patients.

(C) Same as Supplementary Fig. S5c except module preservation was assessed in iPSC-derived motor neurons from ALS8 patients.

(D) Same as Supplementary Fig. S5d except using a dataset of iPSC-derived motor neurons from ALS8 patients. FDR corrected enrichment p-values < 0.05 are shown within the parentheses.

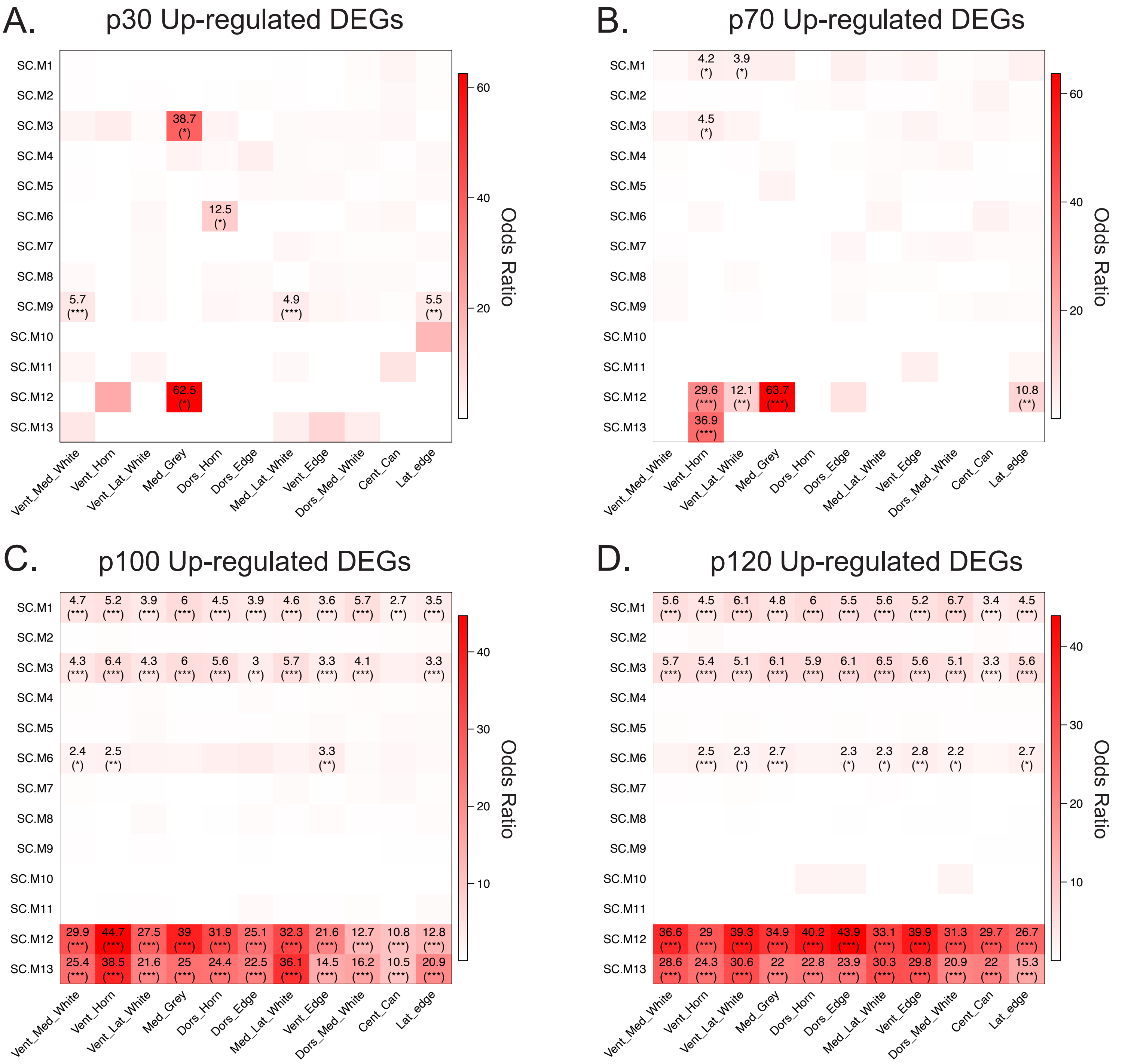
A.
p30 Up-regulated DEGs
B.
p70 Up-regulated DEGs
C.
p100 Up-regulated DEGs
D.
p120 Up-regulated DEGs
SC.M1
SC.M2
SC.M3
SC.M4
SC.M5
SC.M6
SC.M7
SC.M8
SC.M9
SC.M10
SC.M11
SC.M12
SC.M13
Vent_Med_White
Vent_Horn
Vent_Lat_White
Med_Grey
Dors_Horn
Dors_Edge
Med_Lat_White
Vent_Edge
Dors_Med_White
Cent_Can
Lat_edge
Odds Ratio

**Supplementary Figure S8.** Enrichment of ALS up-regulated genes for each Anatomical Annotated Region (AAR) from spatial transcriptomics study using SOD1-G93A mice in co-expression modules.

(A) Enrichment of up-regulated DEGs at stage p30 within co-expression modules. Only enrichments with an FDR corrected p-value < 0.05 are displayed. * (FDR corrected p-value < 0.05). ** (FDR corrected p-value < 0.01). *** (FDR corrected p-value < 0.001).

(B, C, E) Same as (A) except for the p70, p100, and p120 mouse respectively.

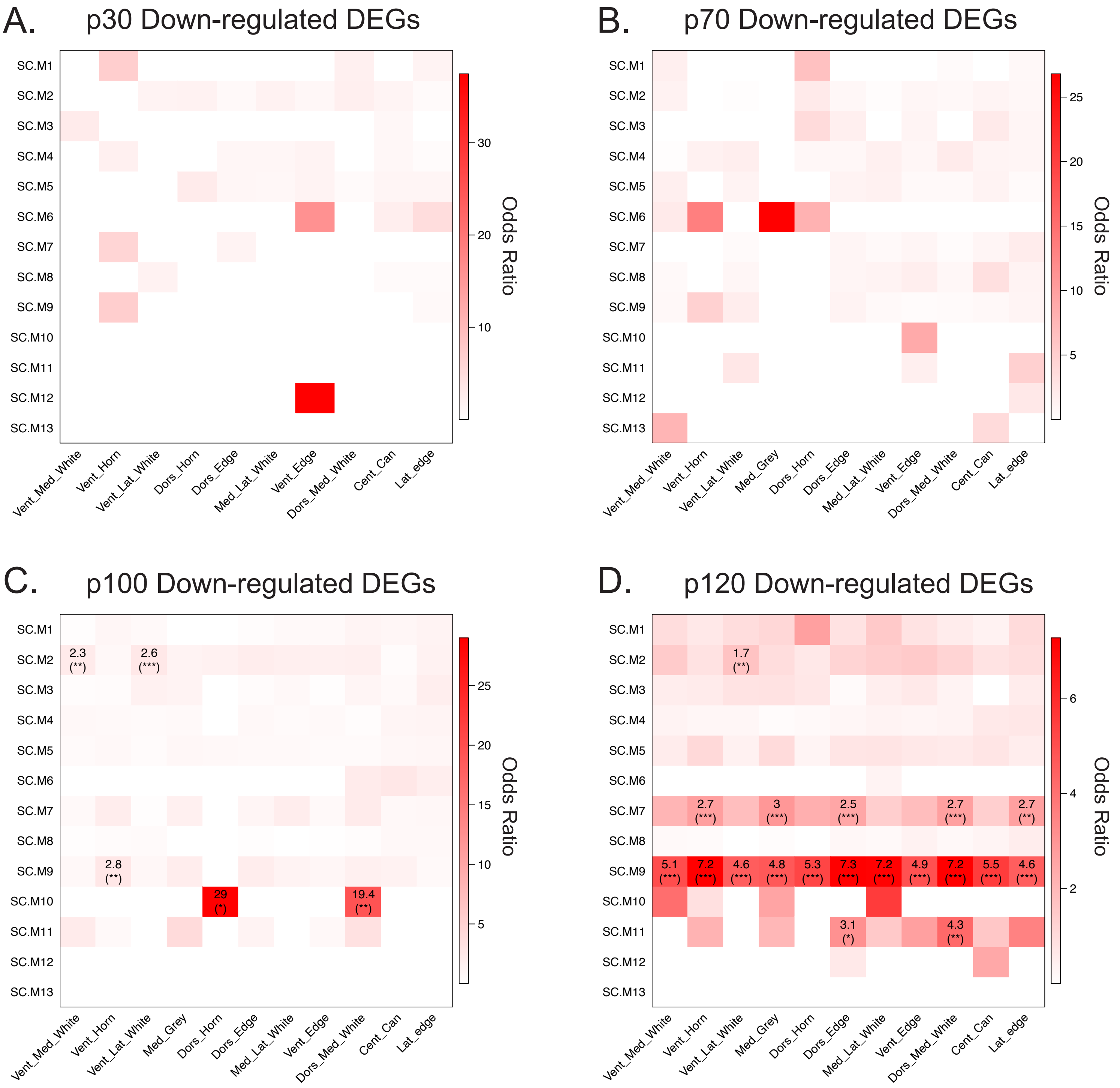
A.
p30 Down-regulated DEGs
SC.M1
SC.M2
SC.M3
SC.M4
SC.M5
SC.M6
SC.M7
SC.M8
SC.M9
SC.M10
SC.M11
SC.M12
SC.M13
Vent_Med_White
Vent_Horn
Vent_Lat_White
Dors_Horn
Dors_Edge
Med_Lat_White
Vent_Edge
Dors_Med_White
Cent_Can
Lat_edge
Odds Ratio
30
20
10
B.
p70 Down-regulated DEGs
SC.M1
SC.M2
SC.M3
SC.M4
SC.M5
SC.M6
SC.M7
SC.M8
SC.M9
SC.M10
SC.M11
SC.M12
SC.M13
Vent_Med_White
Vent_Horn
Vent_Lat_White
Med_Grey
Dors_Horn
Dors_Edge
Med_Lat_White
Vent_Edge
Dors_Med_White
Cent_Can
Lat_edge
Odds Ratio
25
20
15
10
5
C.
p100 Down-regulated DEGs
SC.M1
SC.M2
SC.M3
SC.M4
SC.M5
SC.M6
SC.M7
SC.M8
SC.M9
SC.M10
SC.M11
SC.M12
SC.M13
2.3 (**)
2.6 (***)
2.8 (**)
29 (*)
19.4 (**)
Vent_Med_White
Vent_Horn
Vent_Lat_White
Med_Grey
Dors_Horn
Dors_Edge
Med_Lat_White
Vent_Edge
Dors_Med_White
Cent_Can
Lat_edge
Odds Ratio
25
20
15
10
5
D.
p120 Down-regulated DEGs
SC.M1
SC.M2
SC.M3
SC.M4
SC.M5
SC.M6
SC.M7
SC.M8
SC.M9
SC.M10
SC.M11
SC.M12
SC.M13
1.7 (**)
2.7 (***)
3 (***)
2.5 (***)
2.7 (***)
2.7 (**)
5.1 (***)
7.2 (***)
4.6 (***)
4.8 (***)
5.3 (***)
7.3 (***)
7.2 (***)
4.9 (***)
7.2 (***)
5.5 (***)
4.6 (***)
3.1 (*)
4.3 (**)
Vent_Med_White
Vent_Horn
Vent_Lat_White
Med_Grey
Dors_Horn
Dors_Edge
Med_Lat_White
Vent_Edge
Dors_Med_White
Cent_Can
Lat_edge
Odds Ratio
6
4
2

**Supplementary Figure S9.** Enrichment of ALS down-regulated genes for each Anatomical Annotated Region (AAR) from spatial transcriptomics study using SOD1-G93A mice in co-expression modules.

(A) Enrichment of down-regulated DEGs at stage p30 within co-expression modules. Only enrichments with an FDR corrected p-value < 0.05 are displayed. * (FDR corrected p-value < 0.05). ** (FDR corrected p-value < 0.01). *** (FDR corrected p-value < 0.001).

(B, C, E) Same as (A) except for the p70, p100, and p120 mouse respectively.
